# Supplementary material for: MetaQTL: a package of new computational methods for the meta-analysis of QTL mapping experiments
Source: BMC Bioinformatics. 2007 Feb 8;8:49. doi: 10.1186/1471-2105-8-49 (PMC1808479; doi:10.1186/1471-2105-8-49)
Supplement: Additional File 7 — MetaQTL Package : jar file and tutorial. This Zip archive contains both the MetaQTL JAR file and the files of the tutorial. [file 1471-2105-8-49-S7.zip › org.inra.metaqtl/doc/org/metaqtl/class-use/TreeNode.html]

Uses of Class org.metaqtl.TreeNode


|  |  |  |
| --- | --- | --- |
| **Overview** \| **Package** \| **Class** \| **Use** \| **Tree** \| **Deprecated** \| **Index** \| **Help** | |  |
| PREV   NEXT | **FRAMES**    **NO FRAMES**     **All Classes** |  |


---


## **Uses of Class org.metaqtl.TreeNode**

| Packages that use TreeNode | |
| --- | --- |
| **org.metaqtl** |  |

Uses of TreeNode in org.metaqtl

| Fields in org.metaqtl declared as TreeNode | |
| --- | --- |
| `TreeNode[]` | `TreeNode.children`             Left and right children, or null |
| `TreeNode[]` | `Tree.nodes`             The nodes of the tree. |
| `TreeNode` | `TreeNode.parent`             The parent of the node |
| `TreeNode` | `Tree.root`             The root of the tree. |

| Methods in org.metaqtl that return TreeNode | |
| --- | --- |
| `TreeNode[]` | `TreeNode.getChildren()` |
| `TreeNode` | `Tree.getLeaf(int i)` |
| `TreeNode` | `TreeNode.getParent()` |
| `TreeNode` | `Tree.getRoot()` |

| Methods in org.metaqtl with parameters of type TreeNode | |
| --- | --- |
| `void` | `TreeNode.addChild(TreeNode node)`             Add a child to the node. |

Constructors in org.metaqtl with parameters of type TreeNode

`Tree(TreeNode root)`

`TreeNode(int idx,
TreeNode[] children)`
  
          Creates a new instance of `TreeNode` with the
given indice `idx` and the children of the node.

---


|  |  |  |
| --- | --- | --- |
| **Overview** \| **Package** \| **Class** \| **Use** \| **Tree** \| **Deprecated** \| **Index** \| **Help** | |  |
| PREV   NEXT | **FRAMES**    **NO FRAMES**     **All Classes** |  |


---
